# The Thirteenth Annual Meeting of the Medical Association of the Southwest

**Published:** 1918-09

**Authors:** 


### The Thirteenth Annual Meeting of the Medical Association of the Southwest.

The Thirteenth Annual Meeting of the Medical Association of the Southwest will be held in Dallas, Texas on October 15, 16 and 17, 1918. The medical profession of Texas has invited this society to hold this meeting in Dallas, which city has a reputation for entertaining medical associations, and this in itself, is a guarantee of a large and successful meeting.

Plans are already under way, looking to arranging an exceptionally good program for this convention and "Winning the War," also other subjects at this time, seriously engaging the attention of men belonging to the medical fraternity, will be featured in papers presented before the organization. Plans are being made to have the Government represented with some strong men to present the war side of medicine, and other topics of great interest will be well handled.

Dallas has splendid facilities for offering some exceptionally good clinics at its large hospitals, so that clinics will feature the meeting, and it is probable that the Dallas members of the profession will see to it that visiting physicians who might like to remain over a few days following the close of the convention, will be given an opportunity to do so with additional clinics.

The chairmen of the committees on programs for the different sections are actively at work, soliciting papers for the Dallas meeting.

